# Establishment of a Mesenchymal Stem Cell Bank

**DOI:** 10.4061/2011/905621

**Published:** 2011-08-04

**Authors:** Khushnuma Cooper, Chandra Viswanathan

**Affiliations:** Reliance Life Sciences Pvt. Ltd., Dhirubhai Ambani Life Sciences Centre, R-282, TTC Area of MIDC, Thane-Belapur Road, Rabale, Navi Mumbai, Maharashtra—400701, India

## Abstract

Adult stem cells have generated great amount of interest amongst the scientific community for their potential therapeutic applications for unmet medical needs. We have demonstrated the plasticity of mesenchymal stem cells isolated from the umbilical cord matrix. Their immunological profile makes it even more interesting. We have demonstrated that the umbilical cord is an inexhaustible source of mesenchymal stem cells. Being a very rich source, instead of discarding this tissue, we worked on banking these cells for regenerative medicine application for future use. The present paper gives a detailed account of our experience in the establishment of a mesenchymal stem cell bank at our facility.

## 1. Introduction

Stem cell research and science is likely to offer a second generation of drugs, for hitherto untreatable clinical disorders. Regenerative medicine centers all over the world are working on different cell types to meet this need. The ethics issues and other controversies around the embryonic cells make it a less interesting candidate for clinical applications. Other newer sources of stem cells are induced pluripotent stem cells (iPS cells). The generation of human iPS cells from adult stem cells makes it possible to produce patient-specific ES-like stem cells for therapeutic purposes. But there are several issues on the processes adopted to produce the cells of interest, so it is still in the research level. In sharp contrast, progress with adult stem cells has been impressive. They would have a broader differentiation capacity than previously thought, since they give rise to cell types of multiple tissues [1]. 

Among all adult stem cell types, the bone-marrow-derived mesenchymal stem cells (BMMSCs) have been the most extensively studied cell type so far [2–4]. Bone marrow aspiration is invasive and painful; moreover, cell yield and differentiation potential of MSCs could decline with age [5, 6]. Amongst several other sources studied, umbilical cord blood is another source of mesenchymal stem cells, but the low frequency of MSCs in cord blood makes it less attractive [7–9]. Thus culturing and deriving MSCs from all cord blood units is less interesting and may not be rewarding [10–12]. We have studied umbilical cord tissue which provides a unique source of mesenchymal stem cells (MSCs) with immense potential for tissue repair [13–15]. Collection of umbilical cord is easy, with no ethical concern, and does not harm newborns and the mothers. The plasticity of umbilical cord MSC (UCMSC) has been previously demonstrated by our group [15]. UCMSCs can be expanded, are remarkably stable, and do not trigger any strong immune response. Their immunomodulatory action opens a new direction to possibly reduce the rejection in allogeneic transplantation situations [16]. The immense potentials of these cells for possible future regenerative medicine applications can, therefore, not be overlooked. The plasticity and expandability of these cells at a distant future, even after prolonged cryopreservation, is an additional attraction. All these make UCMSCs bankable and usable. 

We did extensive testing on these cells to ensure their safety, pluripotency, and efficacy after prolonged storage periods. Here, we share our experience on all the aspects of MSC banking.

## 2. Objectives

The main objectives of banking cells from this source were

1. to develop a mesenchymal stem cell bank that would ensure availability of high-quality, reliable-and-well characterized human mesenchymal stem cells for clinical application,
2. to optimize the cell numbers at the master and working cell bank levels,
3. to establish stability studies at different time points during the defined storage period,
4. to establish the functional capability of cells stored for varying periods in liquid nitrogen,

(5) to put in place documentation formats for raw material procurement, manufacturing process, quality control, quality assurance and deviation if any,
(6) establish a comprehensive quality management system to support the cGMP facility requirements throughout the process of cell banking.

## 3. Material and Methods

### 3.1. Informed Consent

The significance of the consenting process cannot be underestimated. An informed consenting procedure was administered to few pregnant women by our trained staff or the obstetricians themselves. At antenatal clinics, these mothers were given a leaflet which explained the cord tissue banking process and the potential use of UCMSC as an alternative for a possible future clinical application. The obstetricians or the trained staff discussed the project with the mother, answering any queries that arose. Each mother responded to questions in a donor evaluation form which was designed to ascertain the risk of transmissible diseases, both infectious and genetic. Parents were given the opportunity to read the information prior to delivery and to make an informed choice to donate the umbilical cord for research. They were given the full liberty to refuse collection. Mothers gave a written consent for the collection of cord tissue postpartum and also gave consent to provide blood samples for all the mandatory tests. The protocol of the informed consent was approved by the Institutional Stem Cell Committee.

### 3.2. Raw Material Collection and Transportation

Cord-tissue-derived stem cell banking basically involves three important steps: collection, processing, and cryopreservation of tissue derived stem cells. 

The cord tissue collection process was relatively simple, safe painless and was done just immediately after child birth. The cord was clamped and cut and 15 cms of the cord was transferred into a labeled tube containing the collection media supplemented with antibiotics. A specialized validated temperature controlled container (2–15°C) was given to those parents who had chosen to donate their child's MSCs for this specific standardization. The collection kit with the cord was transported to the processing facility within 36 hrs. On arrival at the processing facility, the sample was allocated a barcode that enables it to be tracked right throughout processing and banking.

### 3.3. Infectious Disease Testing

Before processing the umbilical cord tissue, the maternal blood was screened for infectious disease markers like HIV I & II and HCV antibodies, HbsAg, Syphilis, and CMV IgG and IgM as per existing regulatory guidelines. If maternal blood was reactive for any of the infectious diseases, it was not taken up for standardization. Increased levels of CMV IgG, were not a disqualification. The cord tissue was then processed for isolation and characterization of stem cells.

### 3.4. Processing Facility

All the samples were processed in the company's GMP-compliant facility comprising of self-contained Grade B cell processing suites. Each processing suite has a Class II microbiological safety cabinet providing the Grade A air environment required for open processing of cell materials. The suites are self-contained and independent of each other in order to minimize the chances of cross-contamination. Each suite is capable of operating independently. All liquid nitrogen storage containers were kept in a separate room along with controlled rate freezers which were used for cryopreservation of the derived UCMSCs.

### 3.5. Cord Tissue Processing

Five cord tissues were used for the standardization process. Only seven to eight cms of the cord was used. Only clean, glistening, nonsuppurative, non contaminated tissues were taken up for processing. The cords were washed with phosphate-buffered saline supplemented with antibiotics. Approximately twenty tissue explants of about 2 mm size each were plated in tissue-culture-grade petri dishes containing DMEM/F12 with fetal bovine serum supplemented with FGF. The dishes were left undisturbed in a 5% CO_2_ incubator maintained at 37°C for 4-5 days after which fresh culture medium was added to the dishes. Adherent cells were allowed to expand for 10–15 days by changing the media at an interval of 2 days. The cells were harvested at 80–90% confluency using TrypLE Select. A cell count was performed after harvesting the cells, and about a million cells were replated into 300 cm^2^ tissue culture flasks for further expansion. All reagents used were quality assured, and, in the house quality assurance approvals were obtained prior to usage as per cGMP guidelines.

### 3.6. Generation of Master and Working Cell Banks

At all steps, a detailed consumption of the media and reagents used for processing was recorded. Appropriate batch numbers, were assigned to the cell banks. In order to ensure supply of adequate cells for future requirement, a master cell bank and working cell bank concept was adopted. This two-tiered cell banking system was also felt so as to optimize the space in the liquid nitrogen storage containers. Thus, in all the cases, a master cell bank was planned from which a working cell bank was to be made for future use. Earlier we have published our data on immunophenotyping, differentiation potential, and karyotyping of UCMSCs expanded till passage 8 that helped us come up with a suitable strategy to bank these cells [15]. Time taken to culture and harvest adequate cell numbers was noted down with the variables that affected the numbers. At all times, storage of the cells was planned in 1.8 mL-capacity cryovials.

### 3.7. Quality Control Tests Performed

All samples underwent the following quality control tests based on available international guidelines: 

(1) cell enumeration (2) phenotypic characterization, (3) viability determination, and (4) microbiological tests for sterility, mycoplasma, endotoxin levels on the cell supernatants.

**(1) Cell Enumeration**

Cell counts were taken using an automated cell counter. For generation of a master cell bank, cells were frozen at passage 1. From the master cell bank, a working cell bank was created to ensure that adequate cells can be further derived as per requirements.

**(2,3) Phenotypic Characterization and Viability Determination**

Cells from each passage were subjected to immunophenotypic analysis. Cultured cells were incubated with various mouse anti-human antibodies conjugated to either FITC, PE, or PerCP. Antibodies used were CD45, CD73, CD105, SSEA-4, HLA-DR, HLA-ABC, CD31, CD14, CD44, CD29 and vWF. While all antibodies were procured from BD Pharmingen, USA, only CD105 antibody was procured from Caltag Laboratories.

After incubation for 20 mins at 4°C, the cells were washed with PBS and acquired using a FACS Calibur flow cytometer (Beckton Dickinson). Cell viability was also determined by staining the cells with 7-AAD (7 Amino Actinomycin D) and analysed using a FACS Calibur flow cytometer. Non-specific fluorescence was determined by staining the cells with directly conjugated isotype-matched anti-mouse monoclonal antibodies. Approximately 10,000 events were acquired and data analysis was performed using the CellQuest software.

**(4) Microbiology Tests**

To detect the presence of aerobic and anaerobic microbes, sterility testing was done on the cell wash sample. This test was performed by inoculating samples in two different sterile nutrient mediums, namely, Fluid Thioglycolate Medium (FTM) and Soybean Casein Digest Medium (SCDM).

The Limulus Amebocyte Lysate (LAL) test is a qualitative test for Gram-negative bacterial endotoxin. Limulus Amebocyte Lysate was reconstituted with LAL reagent water and then mixed in equal parts with the sample. After incubation, and in the presence of endotoxin, gelation occurs; in the absence of endotoxin, gelation does not occur. Sterility and endotoxin testing were performed on the cell wash samples obtained at the time of harvest.

Mycoplasma detection was done by semi quantitative PCR which is the method of choice for detection of mycoplasma contamination in cell cultures. Commercially available kit (Minerva Biolabs, GmbH) was used for the detection of mycoplasma in the cell culture supernatant.

During the banking process, segregation of fully tested samples from untested samples was ensured by using two separate liquid nitrogen storage containers housing “Quarantined” and “Ready for use” samples with the corresponding labels. Quarantined samples are transferred to the final storage after receipt of all the reports. Once the cells are ready for use, a certificate of analysis was generated.

### 3.8. Stability Studies

Stability studies were designed and conducted to determine the optimum period for which the UCMSCs can be cryopreserved without having any effect on viability. For this, UCMSCs were isolated and expanded in cell culture media. After expansion, approximately 1–20 million UCMSCs were frozen per cryovial and stored in liquid nitrogen. Cells were frozen at a controlled rate to minimize freeze-related trauma. Before freezing, the cell count, viability, and immunophenotyping were done. At specified time points (1 month, 6 months, 1 year, 2 years, and 3 years), the cryovials were thawed in a 37°C water bath. Once thawed, the cell count was taken using an automated hematology analyzer. Immunophenotyping and viability assessment were done immediately postthawing using a flow cytometer. Karyotyping was performed on the cells from every passage up to passage 8, and this has already been published earlier [15].

### 3.9. Documentation

Documentation, an integral part of any process, is very important to develop proper processing steps. Using the international guidelines available, all relevant information and tests relating to the raw materials were performed at various stages, and the results thereon were documented for each sample in the batch manufacturing record (BMR). The batch manufacturing record, thus, developed was later approved by the quality assurance team. 

Each sample was labeled with a unique barcode number. The barcode labels withstand the freezing procedure and were validated for use in liquid nitrogen tanks. The location of the cryopreserved samples was maintained in the BMR, and the inhouse developed Regen software helped rapid and accurate retrieval. 

All documents relating to cell bank manufacturing, like batch manufacturing record, cell harvest details, freezing records, phenotypic characterization, viability data, and the details of the master and working cell bank and their location were made available for review by the quality assurance team and then archived for future reference.

## 4. Results

### 4.1. Growth Kinetics and Immunophenotyping

**Figure 1:**
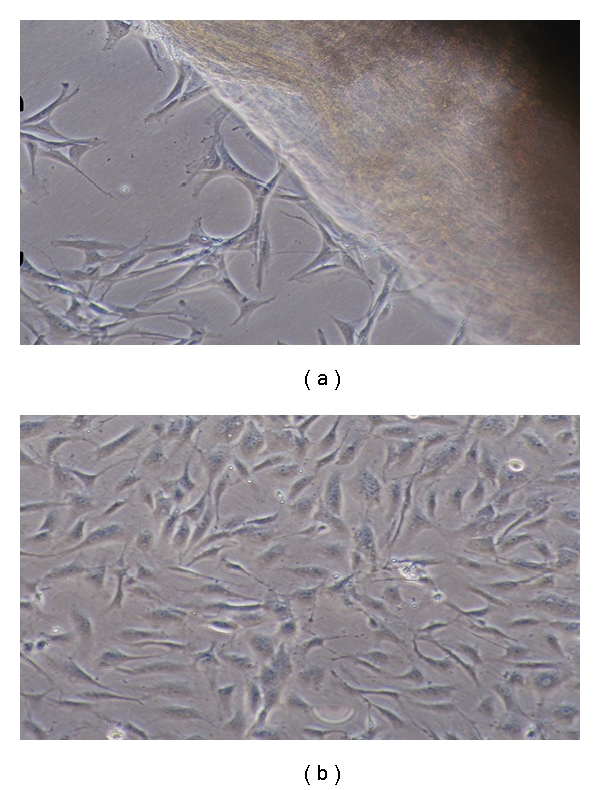
(a) Migration of UCMSCs from the explants after 10–15 days, (b) UCMSCs form a monolayer of adherent fibroblast-like cells by day 25.

**Figure 2:**
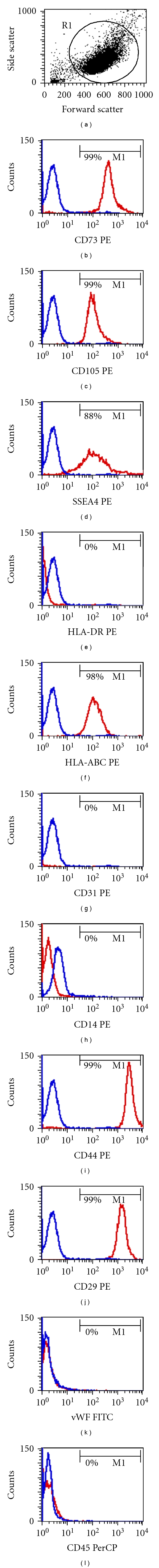
UCMSCs expressed mesenchymal markers and were negative for hematopoietic and endothelial markers.

Umbilical cords from 5 different donors (gestational ages 38–40 weeks) were obtained from maternity hospitals after normal or cesarean deliveries for generation of UCMSCs. All maternal blood samples tested negative for infectious disease markers. MSCs were successfully isolated from all the five cords processed irrespective of the sex of the baby, gestation, and ethnicity (Table 1). Data of cell counts at passage 1 of all the five samples is also shown in Table 1, and it is clear that we could obtain about 40–50 million cells from about 7 to 8 cms of the cord used for processing. UCMSCs started to migrate from the explants within 10 to 15 days and displayed a homogenous fibroblast-like morphology (Figure 1). Flow cytometry studies showed that more than 90% of the UCMSCs displayed uniform mesenchymal stem cell markers such as CD73, CD105, CD44, and CD29. UCMSCs were negative for haematopoietic and endothelial markers such as CD45, CD14, CD31, and vWF. Expression of HLA-DR antigen was not expressed although the cells expressed HLA-ABC antigen. It is important to note that UCMSCs also expressed the stem cell marker SSEA-4 (Figure 2, Table 2). Viability of the cells was determined to be more than 90% by 7-AAD dye exclusion method using flow cytometry.

### 4.2. Quality Control Tests

Creation of a master cell bank was the first step in the establishment of banking. A master cell bank was generated from each case wherein cells were cryopreserved at passage 1 after about 40 days in culture. Cells were cryopreserved at a concentration of 1-2 × 10^6^/mL in 1.8 mL cryovials. Approximately 10–15 vials were made per sample, and one vial from the master cell bank was allocated for quality control testing such as immunophenotyping and viability studies. All the cells used for generation of master cell bank were free of microbial and mycoplasma contamination.

### 4.3. Cryopreservation and Documentation

UCMSCs that were stored in liquid nitrogen containers provided a secure storage environment and maintained temperatures of −196°C satisfactorily. The liquid nitrogen container was connected to a data logger which recorded the storage temperature in a dynamic mode. UCMSCs were frozen till the temperature reached −150°C before placing into liquid nitrogen container. Freezing records were maintained satisfactorily for all the samples. At the time of release from quarantine, the cells were certified as being sterile, free from mycoplasma and endotoxin.

### 4.4. Stability Studies

**Figure 3:**
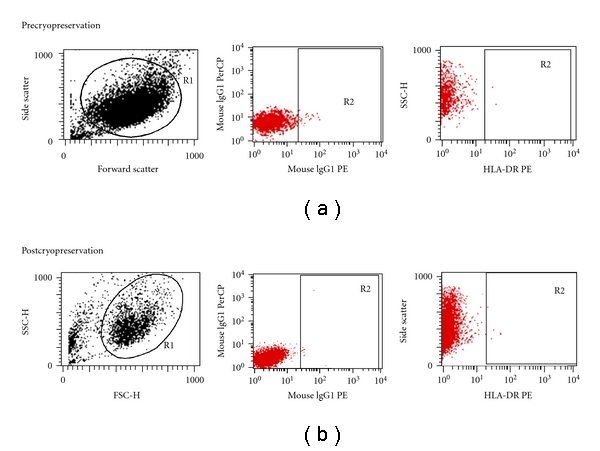
UCMSCs did not show expression of HLA-DR pre- and postcryopreservation.

UCMSCs with a pre-freeze viability of 98-99% and biomarker expression of 98% were stored in liquid nitrogen for a period of 1 month, 6 months, 1 year, 2 years, and 3 years. Upon thawing, the cell count, viability, and immunophenotyping performed at every time point up to three years are shown in Table 3. UCMSCs cryopreserved for 3 years in liquid nitrogen showed a negligible drop in the cell count and viability as compared to the earlier time points (Table 3). UCMSCs did not express HLA-DR antigen pre- and postthawing (Figure 3) at all time points.

## 5. Discussion

MSCs are multipotent cells that have the ability to give rise to different cell types in the human body. They have a very vast potential to serve as a new research tool to support clinical applications for the future. They have been isolated by various groups from different regions of the cord, namely, the perivascular region, the intervascular region, and also the subamnion. Sarugaser et al. reported the isolation of mesenchymal stem cells from the perivascular region of the umbilical cord [17] whereas Covas et al. reported the isolation of mesenchymal stem cells from the umblical vein [18]. In our study, we have isolated MSCs having fibroblast-like morphology and expressing cell surface markers such as CD73, CD105, CD44, and CD29 from Wharton's jelly portion of the umbilical cord tissue. We have also shown that UCMSCs expanded in culture remain viable in liquid nitrogen even after cryopreservation for up to 3 years although studies on prolonged cryopreservation periods are proposed to be continued. 

Several studies, including our own, have shown that MSCs are immunomodulatory in nature and are expected to play a major role in transplantation biology opening up new promising avenues for them to be used in the allogeneic settings [16, 19–21]. 

Due to their immunomodulatory properties, in addition to their regenerative potential, MSCs are currently being explored in other therapeutic approaches such as to improve haematopoietic reconstitution after HSCT and to overcome GVHD upon allogeneic transplantation [22, 23]. Le Blanc et al. [23] have reported on the use of haploidentical MSC in conjunction with allogeneic hematopoietic stem cell transplantation to enhance engraftment. All patients achieved neutrophil and platelet engraftment and 100% donor chimerism. Osiris therapeutics have conducted a phase 1 clinical trial to study the effect of MSCs on haematopoietic stem cell engraftment. The MSC infusions were well tolerated, and there were no drug-related serious adverse events and all patients achieved neutrophil engraftment [24]. Both of these studies show the safety of MSC infusion during hematopoietic stem cell transplantation. Osiris therapeutics is also conducting clinical trial using MSCs (Prochymal) for treatment of resistant Crohn's disease which is currently ongoing [25]. Hemorrhagic cystitis is associated with HSCT, and its incidence varies from <10% to more than 70%. Ringdén et al. [26] have used MSCs to heal such therapy-induced tissue toxicity. In five of the seven patients, the severe hemorrhagic cystitis cleared after MSC infusion, and gross hematuria disappeared after median 3 (1–14) days. This study shows that MSCs is a novel treatment that may be used for GVHD, tissue toxicity, and hemorrhages because of its immune inhibitory and anti-inflammatory effects. MSCs have also been used for regeneration of the myocardium after myocardial infarction [27, 28]. We have previously reported our findings on the use of MSCs in patients undergoing coronary bypass surgery [28]. Our study showed that transepicardial injections of MSCs were safe and feasible, correction of perfusion defect was also observed although no significant change was observed in the ejection fraction. Chen et al. [29] have also reported improvement in cardiac function of infarcted myocardium with intracoronary transplantation of human mesenchymal stem cells identified by noninvasive and cardiac electromechanical mapping. Williams et al. [30] have shown that transcatheter, intramyocardial injection of autologous bone marrow preparations in patients with chronic ischemic cardiomyopathy was welltolerated and produced functional recovery in scarred myocardium and reverse remodeling of the LV chamber. Encouraging results have also been seen when MSCs were used for neuronal treatment [31]. Venkataramana et al. [32] have reported a clinical study in which seven Parkinson's disease patients were transplanted with MSCs into the sublateral ventricular zone by stereotaxic surgery. Results showed that injection of MSCs was safe, and no serious adverse events occurred after stem cell transplantation. MSCs have also been used for treatment of osteogenesis imperfecta by Horwitz et al. [20]. 

A well-designed manufacturing record helps with ready retrieval of the banked units and locating the position of the units, besides helping us to capture all the intricate data during the entire culturing process. This will help designating specific storage location for each unit which in the future will prevent wrong units from being retrieved. Such details are also available in our locally designed software Regen. Adequate care is taken to prevent cross-contamination, and mixup as per the guidelines in force for cell therapy manufacture. 

The establishment of various processes and procedures in the setting up of the experimental bank has been worthwhile. While a detailed costing is being done, it appears that the expenses involved in maintenance of cell cultures up to passage 1, transportation, infectious disease testing, and cryopreservation would likely be in the range of $1000–1200, which excludes infrastructure, maintenance, and manpower cost. 

We have shown that appropriate maintenance of biological characteristics and uniformity of the cells is possible using traditional cryopreservation methods. Our studies have demonstrated that it is possible to generate and bank large numbers of UCMSCs for regenerative medicine applications for future use. The establishment of a stem cell bank having well-characterized and “ready-to-use” allogeneic MSCs could be promising and attractive to clinical researchers.

## 6. Conclusion

Our results show that simple and appropriate validated protocols for cord tissue processing under good manufacturing conditions can help in the development of cost-effective banking of UCMSCs.

## Figures and Tables

**Table 1:** Parameters of all the five cords studied.

Sample no.	Mothers age	Sex of baby	Gestation (weeks)	Cell count at P1 (10^6^)
(1)	25	Female	40	45
(2)	39	Male	39	50
(3)	32	Male	38	42
(4)	24	Female	38	40
(5)	28	Male	40	47

**Table 2:** Range and median of surface marker expression of UCMSC as detected by flow cytometry. Data of all five cords processed.

CD Markers	Range (%)	Median (%)
CD73	99.11–99.79	99.46
CD105	99.81–100	99.95
CD44	99.32–100	99.57
CD29	95.47–99.4	99.90
CD45	0.15–2.15	0.2
CD14	0–0.9	0.24
CD31	0–0.63	0.08
vWF	0–3.23	0.14
HLA-DR	0.24–2.3	0.42
HLA-ABC	75.62–99.82	99.46
SSEA-4	45.96–94.38	76.6

**Table 3:** Stability data of UCMSCs from all five cords at different time points.

Sample no.	Parameters	Time points					
		Initial	1 month	6 months	1 year	2 years	3 years
Sample 1	Cell count (10^6^)	12.0	11.8	11.2	11.1	11.6	10.8
	Viability (%)	98.9	98.3	98.1	98.8	97.7	98.3
	Immunophenotype (CD73 & CD105) (%)	98.0	98.0	98.0	98.0	98.0	98.0

Sample 2	Cell count (10^6^)	20	19.7	19.4	19.5	19.2	19.1
	Viability (%)	99.5	99.2	99.2	99.0	98.9	98.9
	Immunophenotype (CD73 & CD105) (%)	98.0	98.0	98.0	98.0	98.0	98.0

Sample 3	Cell count (10^6^)	5.0	5.0	4.8	4.6	4.7	4.5
	Viability (%)	99.8	99.7	99.8	99.6	99.5	99.5
	Immunophenotype (CD73 & CD105) (%)	98.0	98.0	98.0	98.0	98.0	98.0

Sample 4	Cell count (10^6^)	1.0	1.0	1.0	1.1	1.0	1.0
	Viability (%)	98.7	98.7	98.5	98.6	98.5	98.5
	Immunophenotype (CD73 & CD105) (%)	98.0	98.0	98.0	98.0	98.0	98.0

Sample 5	Cell count (10^6^)	15.5	15.2	15.0	15.0	15.1	15.0
	Viability (%)	98.0	98.1	97.8	97.8	97.6	97.5
	Immunophenotype (CD73 & CD105) (%)	98.0	98.0	98.0	98.0	98.0	98.0
